# Associations of dietary copper intake with cardiovascular disease and mortality: findings from the Chinese Perspective Urban and Rural Epidemiology (PURE-China) Study

**DOI:** 10.1186/s12889-023-17441-6

**Published:** 2023-12-18

**Authors:** Xiaocong Li, Mahshid Dehghan, Lap Ah Tse, Xinyue Lang, Sumathy Rangarajan, Weida Liu, Bo Hu, Salim Yusuf, Chuangshi Wang, Wei Li

**Affiliations:** 1 Medical Research and Biometrics Center, National Clinical Research Center for Cardiovascular Diseases, Fuwai Hospital, National Center for Cardiovascular Diseases, Peking Union Medical College and Chinese Academy of Medical Sciences, Beijing, China; 2 Population Health Research Institute, McMaster University and Hamilton Health Sciences, Hamilton, ON Canada; 3 JC School of Public Health and Primary Care, The Chinese University of Hong Kong, Hong Kong, Hong Kong SAR, China; 4 State Key Laboratory for Complex, Severe, and Rare Diseases, Peking Union Medical College Hospital, Beijing, 100730 China

**Keywords:** Dietary copper intake, Cardiovascular disease, Mortality

## Abstract

**Background:**

Previous in vitro and animal experiments have shown that copper plays an important role in cardiovascular health. Dietary copper is the main source of copper in the human body and the association between dietary copper and cardiovascular disease remains unclear. Our study aimed to investigate the associations of dietary copper intake with the risk of major cardiovascular disease incidence, cardiovascular disease mortality, and all-cause mortality in Chinese adults.

**Methods:**

Our study is based on Prospective Urban Rural Epidemiology China (PURE-China), a large prospective cohort study of 47 931 individuals aged 35–70 years from 12 provinces in China. Dietary intake was recorded using a validated semi-quantitative food frequency questionnaire designed specifically for the Chinese population. The daily intake of copper was obtained by multiplying the daily food intake with the nutrient content provided in the Chinese Food Composition Table (2002). Cox frailty proportional hazards models were developed to evaluate the association between dietary copper intake with mortality, major cardiovascular disease events, and their composite.

**Results:**

A total of 45 101 participants (mean age: 51.1 ± 9.7 years old) with complete information were included in the current study. The mean dietary copper intake was 2.6 ± 1.1 mg/d. During the 482 833 person-years of follow-up, 2 644(5.9%) participants died, 4 012(8.9%) developed new cardiovascular diseases, and 5 608(12.4%) participants experienced the composite endpoint. Compared with those in the first and second quartile of dietary copper intake, individuals in the third and fourth quantile had higher risk of composite outcomes, all-cause death, cardiovascular disease death, major cardiovascular disease and stroke occurrences. The associations remained similar in the subgroup and sensitivity analyses.

**Conclusions:**

Our findings demonstrated that excessive dietary copper intake was associated with higher risks of death and cardiovascular diseases in Chinese adults. Further studies in populations with different dietary characteristics are needed to obtain dose–response relationships and to refine global dietary recommendations.

**Supplementary Information:**

The online version contains supplementary material available at 10.1186/s12889-023-17441-6.

## Background

According to the World Health Organization, 17.9 million people worldwide die from cardiovascular disease (CVD) every year, accounting for about 32% of all deaths [1, 2]. The 2021 China Cardiovascular Health and Disease Report shows that approximately 330 million patients currently suffer from CVD in China, equivalent to 46.74% and 44.26% of all-cause deaths of 2019 in rural and urban areas, respectively [3]. CVD has been a serious public health problem in China and globally.

Copper plays a vital part in many life processes such as energy metabolism, iron uptake, and signaling in eukaryotic organisms [4]. Excessive copper ions will bind to mitochondrial proteins, causing mitochondrial damage, and resulting in proteotoxic stress-mediated cell death, while insufficient copper in the body may induce arrhythmias, glucose intolerance, hypercholesterolemia and so on [5, 6]. The previously reported basic levels for blood copper were within the range of 0.7 to 1.9 mg/mL [7–9]. Cuproptosis may lead to the occurrence of various diseases, including Wilson’s disease, neurodegenerative diseases, cancer as well as CVDs. As copper cannot be synthesized and stored in the body, the daily intake is the main source of serum copper to supply our demands. Being a potential modifiable risk factor for CVDs, proper amount of copper intake from diet should be recommended to maintain an optimal serum copper level, however, the threshold value remains unclear [10]. It is of great significance to characterize the dose–response relationship between dietary copper intake and CVD incidence or mortality for better prevention and management of CVD and its related diseases.

The results of prior studies regarding the association of dietary copper intake with CVDs and death are inconsistent. The National Health and Nutrition Examination Survey (NHANES) collected the dietary copper intake of study participants through a 24-h dietary recall method. Several studies including a case–control study, cross-sectional study, and propensity score-matched analysis based on this database showed that higher dietary copper intake was associated with lower risk of stroke, myocardial infarction (MI), CVDs, and CVD death [11–14]. A prospective cohort study in 58 646 healthy Japanese applied a validated self-administered food frequency questionnaire to calculate the dietary copper intake and found that dietary copper intake was not associated with coronary heart disease mortality, but was positively associated with mortality from total CVD, stroke, and other CVD deaths [15]. The China Health and Nutrition Survey, indicated a J-shaped association between dietary copper intake and mortality [16]. A cohort study conducted in 1054 older British people also showed a significantly negative association between total mortality and baseline dietary copper intake [17]. At present, epidemiological studies on dietary copper and the risk of CVDs and mortality are still inconsistent, and there has been few large cohort studies to explore the relationship between copper intake and CVDs in China.

Dietary copper mainly comes from shellfish, animal liver and kidney, nuts, cereal germ, and beans and Chinese residents mainly consume copper through meat and meat products, tea, nuts and seeds, and beans [18, 19]. The Prospective Urban Rural Epidemiology (PURE) China study used a validated food frequency questionnaire (FFQ) covering all aspects of diets (including the aforementioned items) to collect dietary data, which is comprehensive enough to estimate the copper intake among Chinese residents and provides us an opportunity to examine the effects of dietary copper on cardiovascular health [20]. Our study aims to explore the association between dietary copper intake and the risk of major CVD incidence, CVD mortality, and all-cause mortality based on dietary data collected from the PURE-China cohort study.

## Methods

### Study design and participants

The PURE study is a large-scale ongoing longitudinal study involving 25 countries from low -, middle-, and high- income countries since 2003 [21, 22]. The PURE-China is the largest site of PURE study recruited 47 931 baseline participants from 115 communities (70 urban and 45 rural) in 12 provinces between 2005 and 2009. The details about study design and participant enrollment have been described elsewhere [21, 23, 24]. Briefly, the locations of PURE-China were grouped into 3 regions: Eastern regions (Beijing, Jiangsu, Shandong, and Liaoning), Central regions (Shanxi, Jiangxi, and Inner Mongolia), and Western regions (Yunnan, Qinghai, Shaanxi, Xinjiang, and Sichuan). Considering the level of economic development and the possibility of long-term follow-up, a three-level cluster sampling (province, community, and household) was used for the selection of the potential participants. All individuals enrolled in the study provided written informed consent, and the study protocol was reviewed and approved by the institutional review board at Fuwai Hospital of the Chinese Academy of Medical Sciences and Beijing Hypertension League Institute.

**Fig. 1:**
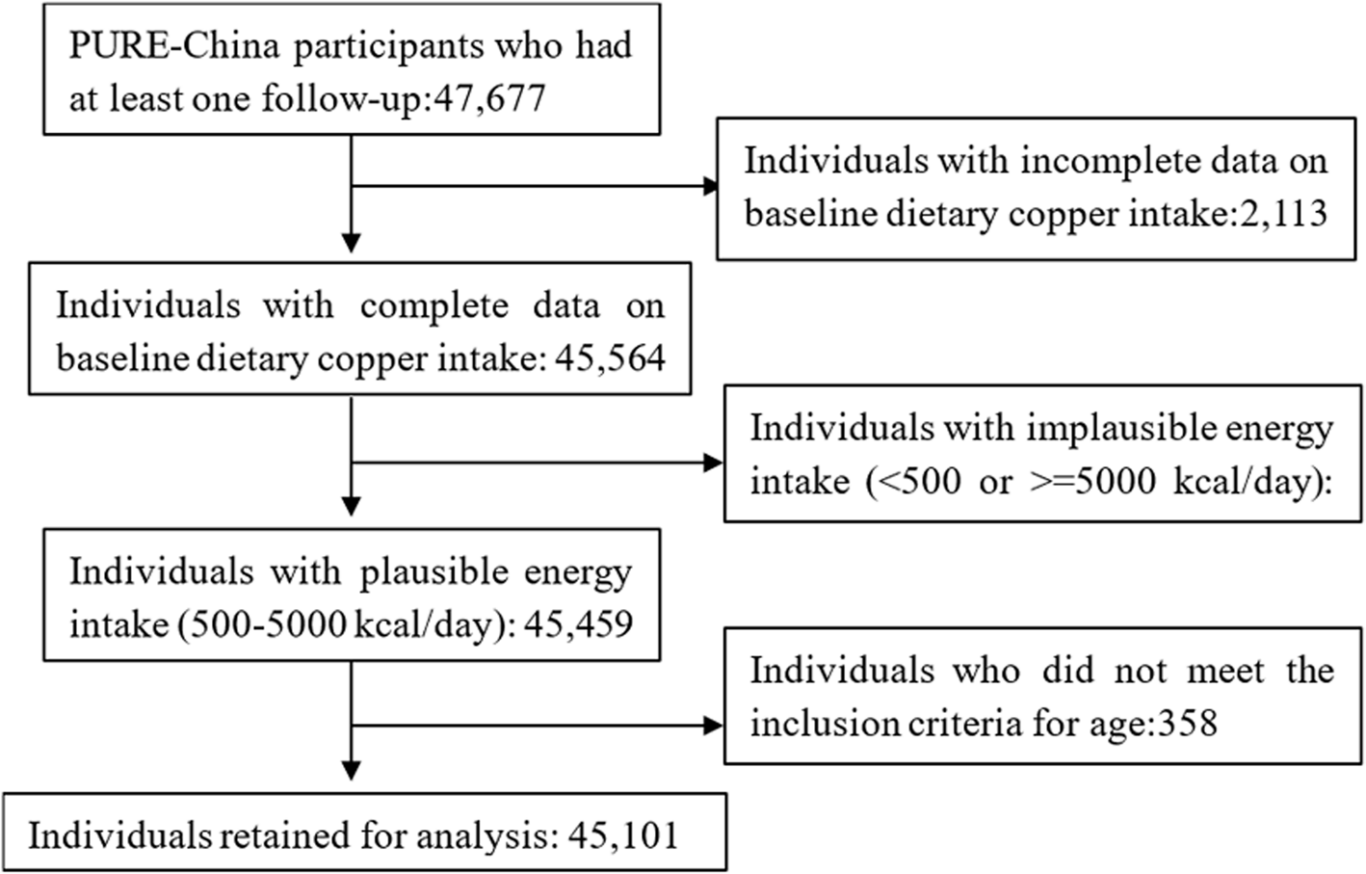
Eligible participants and those included in the analyses of the association between dietary copper intake and health outcomes in PURE-China

All participants were followed up by the trained research team by face-to-face interviews or telephone calls until 15/09/2022 to obtain CVD events and underlying cause of death. Considering our research objectives, we included subjects with at least one follow-up and complete copper intake data in the study, excluding individuals with unreasonable energy intake (< 500 or ≥ 5000 kcal/day) and baseline age not in the range of 35 to 70 years old. Finally, a total of 47 677 participants recruited at baseline had at least one follow-up. 45 564 people had complete data on baseline dietary copper intake, of which 45 459 had plausible energy intake (500–5000 kcal/day). 358 participants were excluded because they did not meet the inclusion criteria for age (as shown in Fig. 1).

### Data collection

Standardized questionnaires and a physical examination were performed at baseline to collect the demographic factors (age, sex), sociodemographic factors (location, education, and occupation), lifestyle factors (smoking, alcohol use, physical activity, and dietary intake), and health-related variables (anthropometric measurements, health history and medication use, and family history of cardiovascular disease).

The location was divided into urban and rural residences. Education status was categorized into primary or less, secondary school, and college/trade school/university education. Occupation was classified as professionals/managers, skilled workers, unskilled workers, and homemakers. Smoking and alcohol consumption status was categorized into smoker or drinker if an individual was currently using or had ever used any tobacco or alcohol products, otherwise they were classified as a non-smoker or non-alcohol drinker. The long-form International Physical Activity Questionnaire (IPAQ) was employed to evaluate the intensities of main types of physical activity. According to the calculated total physical activity, individuals’ physical activity was reported as low, moderate, or high level. Anthropometric measurements included standardized measurements of weight, height, waist to hip ratio (WHR), body mass index (BMI). BMI was calculated as weight in kilograms divided by height in meters squared, and WHR was calculated as waist circumference divided by hip circumference (cm). Hypertension was defined as having a diagnosis of hypertension, taking blood pressure-lowering treatment, or having an average systolic blood pressure of at least 140 mmHg or an average diastolic blood pressure of at least 90 mmHg (or both). History of diabetes was defined as self-reported diabetes or taking diabetes-related medications.

The habitual dietary intake was recorded using a semiquantitative FFQ which was specifically designed for the Chinese population and has been validated in previous studies. The FFQ was administered to Chinese residents with multiple 24-h dietary recalls as the reference method and showed a general well performance [20, 25]. The Chinese-FFQ contains 149 food items, including staple foods, vegetables, fruits, snacks and nuts, legumes, unprocessed/processed meats, fish and other sea foods, eggs, dairy, cooking oil, and condiments. Open-ended response options for consumption frequency (daily, weekly, monthly, annual, or never) and average consumption (Liang, equal to 50 g) of each food were designed to compute the daily dietary intake over the previous year. Daily intakes of energy and all nutrients were obtained by multiplying daily food intake with energy and nutrient contents obtained from the China Food Composition Table (2002). The daily copper intake was estimated by the summation of the copper content in different foods (mg/100 g) multiplied by the intake of that food, which is the same for all macro and micro-nutrients.

### Outcomes

The primary outcome was the composite of all-cause mortality (including CVD mortality and non-CVD mortality) and non-fatal cardiovascular events (including non-fatal MI, stroke, and heart failure (HF)). Secondary outcomes include CVD mortality, all-cause mortality, MI, stroke, and HF. Standard case report forms were used to record data on major cardiovascular events and mortality during follow-up. Any related medical records or certificates were asked to be provided. The ascertainment of clinical events was centrally adjudicated by a trained clinical event committee (CEC) with the use of standardized definitions according to the case-report forms, death certificates, medical records, and verbal autopsies. All outcome events were adjudicated according to the standard predefined definitions and the International Classification of Diseases (ICD)-10 code [26]. And the accuracy of the event report has been assessed in a small sample, confirming that the event reports during central adjudication have sufficient confirmation rate [27]. In the current analysis, we included all adjudicated events in the PURE study database until September 15, 2022.

### Statistical analysis

Counts (percentages) for categorical variables and means (standard deviation, SD) or medians (interquartile range, IQR) as appropriate for continuous variables were used to describe the baseline characteristics for the entire cohort and stratified by copper intake quartiles. Proportions and means of participants' characteristics across different copper intakes were compared by chi-square test and Student T-test or Kruskal–Wallis H test as appropriate. Restricted cubic splines (RCS) with five knots at the 5th, 25th, 50th, 75th, and 95th centiles were performed to test for linearity and explore the shape of association between copper intake and the clinical outcomes.

The association between dietary copper intake and clinical outcomes was assessed using Cox proportional hazards frailty models with a random intercept of center-level clustering. The proportional hazard assumptions were assessed with log–log plots, and the analysis detected no violation. Covariates were identified based on previous literature and proposed mechanisms. Adjusted hazard ratios (HRs) and corresponding confidence intervals (CIs) were computed in the models. In minimally adjusted model, age, sex, and center (as random effects) were included. Then the demographic data; location(urban/rural), education, occupation, and marriage, the lifestyle covariates (including smoking status, alcohol use, and physical activity), baseline health conditions (including BMI, history of diabetes, hypertension, and history of CVDs) and total energy intake were included in the fully adjusted model.

Interaction between dietary copper intake and other covariates (sex, age, BMI, location, and hypertension) was assessed by introducing a multiplication term in the model. And stratified analysis was used to test the consistency of associations between dietary copper intake and the clinical outcome between subgroups. In order to explore the consistency of the association in different geographical regions, we also conducted subgroup analysis by dividing them into three groups: Eastern, Central and Western regions. Participants with events occurring during the first 2 years of follow-up were excluded from the sensitivity analysis to minimize the potential for reverse causation. The analyses by excluding participants who had a history of CVD (stroke, angina, heart attack, coronary artery disease, HF, or other heart diseases) at baseline were also performed as sensitivity analyses. And we also excluded patients with CVD disease history, diabetes and hypertension for further analysis. In addition, we supplemented the main model with adjustments for dietary data, including some macro and micro-nutrients (e.g. protein, lipid, carbohydrate, fiber, zinc, iron, sodium, potassium, and magnesium). Two-tailed statistical tests with *P* < 0.05 were considered statistically significant and all statistical analyses were conducted using SAS 9.4.

## Results

A total of 45,101 participants with complete data on dietary intake and plausible energy intake at baseline were included in the current study with an average age of 51.1 ± 9.7 years old. 18 847(41.8%) of the participants were male, and 23 201(51.4%) participants were from rural areas. The median (IQR) was 2.45 (1.85–3.19) mg/d. Baseline characteristics of the study participants based on quartiles of dietary copper intake are presented in Table 1. The participants with higher dietary copper intake tended to be young men and were more likely to be current smoker and drinker. Homemakers tends to take a lower copper intake. Participants who consume more copper from their diet often have higher levels of physical activity and total energy intake. After a median of 11.9 years of follow-up, 5 608(12.4%) composite outcomes, including 2 644(5.9%) death and 4 012(8.9%) major cardiovascular events happened.

**Table 1:** Participants’ baseline characteristics by quartiles of dietary copper intake

Variables	Total (*N* = 45 101)	< 1.85 mg/d (*N* = 11 159)	1.85–2.45 mg/d (*N* = 11 249)	2.45–3.19 mg/d (*N* = 11 297)	> 3.19 mg/d (*N* = 11 396)
Copper, mg/d, mean ± SD	2.6 ± 1.05	1.5 ± 0.28	2.1 ± 0.17	2.8 ± 0.21	4.1 ± 0.78
Age, year, mean ± SD	51.1 ± 9.65	52.3 ± 9.76	51.6 ± 9.65	50.9 ± 9.59	49.5 ± 9.39
Men, N (%)	18 847(41.8)	3 524(31.6)	4 333(38.5)	5 012(44.4)	5 978(52.5)
BMI, kg/m^2^, mean ± SD	24.6 ± 3.66	24.3 ± 3.67	24.5 ± 3.65	24.7 ± 3.7	24.7 ± 3.62
Rural, N (%)	23 201(51.4)	5 924(53.1)	5 594(49.7)	5 650(50)	6 033(52.9)
Married, N (%)	42 475(94.4)	10 261(92.2)	10 581(94.2)	10 685(94.8)	10 948(96.2)
Education, N (%)					
Low	15 391(34.2)	4 403(39.6)	3 810(34)	3 563(31.6)	3 615(31.8)
Medium	22 959(51)	5 483(49.3)	5 697(50.8)	5 888(52.2)	5 891(51.8)
High	6 629(14.7)	1 229(11.1)	1 713(15.3)	1 827(16.2)	1 860(16.4)
Occupation, N (%)					
Professional/managers	5 680(12.6)	1 175(10.6)	1 520(13.6)	1 542(13.7)	1 443(12.7)
Skilled workers	1 5623(34.8)	3 609(32.5)	3 978(35.5)	4 012(35.6)	4 024(35.4)
Unskilled workers	1 6014(35.6)	3 618(32.6)	3 631(32.4)	4 086(36.3)	4 679(41.2)
Homemaker	7 628(17)	2 710(24.4)	2 078(18.5)	1 631(14.5)	1 209(10.6)
Current Smoker (%)	12 198(27.5)	2 374(21.8)	2 912(26.3)	3 184(28.6)	3 728(33.1)
Current Drinker (%)	10 924(24.5)	2 095(19.2)	2 587(23.2)	2 875(25.7)	3 367(29.9)
Physical activity, N (%)					
Low	6 889(15.5)	2 060(18.6)	1 637(14.8)	1 623(14.6)	1 569(14)
Moderate	1 8781(42.2)	4 976(45)	4 828(43.6)	4 601(41.3)	4 376(39.1)
High	1 8791(42.3)	4 011(36.3)	4 616(41.7)	4 927(44.2)	5 237(46.8)
Hypertension, N (%)	18 713(41.6)	4 500(40.4)	4 628(41.2)	4 837(42.9)	4 748(41.8)
History of diabetes, N (%)	3 779(8.4)	1 145(10.3)	966(8.6)	886(7.8)	782(6.9)
History of CVD, N (%)	3 791 (8.4)	954 (8.5)	993 (8.8)	998 (8.8)	846 (7.4)
Energy, kcal/day, mean ± SD	1964 ± 667	1346 ± 394	1766 ± 426	2058 ± 441	2672 ± 559
Composite outcome, N (%)	5 608(12.4)	1 353(12.1)	1 343(11.9)	1 434(12.7)	1 478(13.0)
CVD incidence	4 012(8.9)	918(8.2)	927(8.2)	1 051(9.3)	1 116(9.8)
Stroke incidence	2 811(6.2)	634(5.7)	633(5.6)	755(6.7)	789(6.9)
MI incidence	1 119(2.5)	267(2.4)	289(2.6)	269(2.4)	294(2.6)
HF incidence	314(0.7)	72(0.6)	63(0.6)	80(0.7)	99(0.9)
All-cause mortality	2 644(5.9)	722(6.5)	643(5.7)	634(5.6)	645(5.7)
CVD mortality	955(2.1)	273(2.4)	201(1.8)	236(2.1)	245(2.1)

*BMI* Body mass index, *CVD* Cardiovascular disease, *HF* Heart failure, *MI* Myocardial infarction

**Fig. 2:**
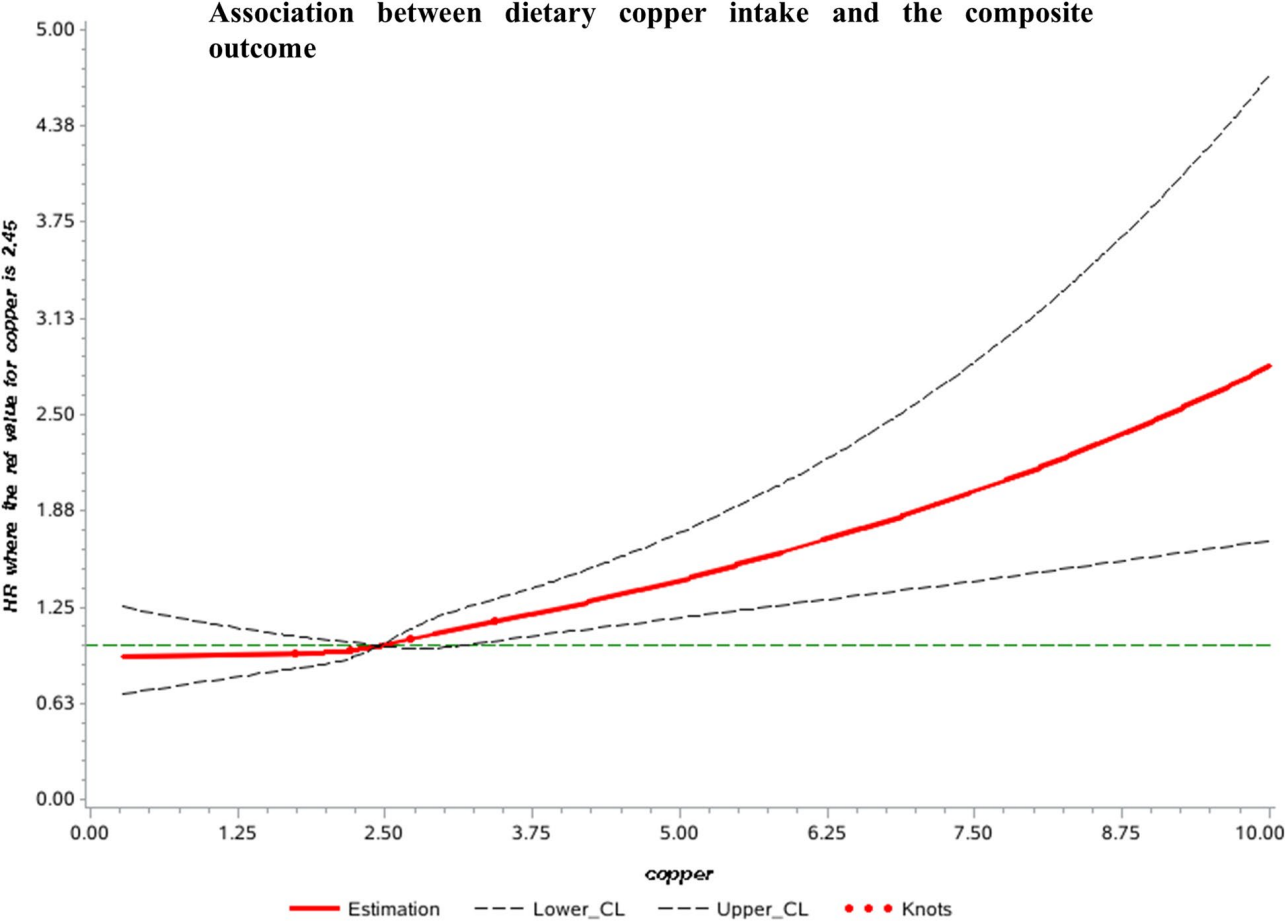
Restricted cubic spline plots between dietary copper intake and the composite outcome

We explored the dose–response relationship between dietary copper intake with CVDs and death using restricted cubic splines (as shown in Fig. 2 and Supplemental eFigure 1). The results suggested a linear association between dietary copper intake of more than 2.45 mg/d and the composite outcome (P for nonlinearity test > 0.05). As shown in Table 2, the age and sex-adjusted model showed insignificant associations between dietary copper intakes with health outcomes. However, after additional adjustment for other CVD risk factors, compared with those in the lowest quartile of dietary copper intake, the risk of the composite outcome was significantly higher (HR, 1.10; 95% CI, 1.00–1.22 for the third quartile; HR, 1.17; 95% CI, 1.02–1.34 for the highest quartile; P for trend = 0.01). For individual outcomes, dietary copper intake of more than 2.45 mg/d was positively associated with of all-cause mortality, CVD mortality, major CVD risk and stroke risk. There was no significant association between copper intake and risk of HF and MI incidence. Further adjustments for the intakes of macronutrients and micronutrients such as protein, lipid, carbohydrate, fiber, zinc, iron, sodium, potassium, and magnesium did not change the results (Table 2).

**Table 2:** The relationship between intake of copper and health outcomes ^a^

	Dietary copper intake				*P* for trend
	HR (95%CI)				
	< 1.85 mg/d	1.85–2.45 mg/d	2.45–3.19 mg/d	> 3.19 mg/d	
Composite outcome					
Minimal model	1.00 (reference)	0.95(0.88,1.02)	0.98(0.90,1.05)	0.98(0.91,1.06)	0.8580
Main model	1.00 (reference)	1.02(0.94,1.12)	1.10(1.00,1.22)^b^	1.17(1.02,1.34)^b^	0.0148^b^
Main model + Nutrients	1.00 (reference)	1.01(0.92,1.11)	1.07(0.96,1.20)	1.10(0.94,1.29)	0.1756
CVD incidence					
Minimal model	1.00 (reference)	0.95(0.87,1.05)	1.01(0.92,1.10)	1.03(0.93,1.13)	0.3675
Main model	1.00 (reference)	1.03(0.92,1.14)	1.14(1.01,1.29)^b^	1.25(1.06,1.47)^b^	0.0038^b^
Main model + Nutrients	1.00 (reference)	1.00(0.90,1.12)	1.10(0.96,1.25)	1.15(0.95,1.39)	0.1004
Stroke incidence					
Minimal model	1.00 (reference)	0.93(0.83,1.04)	1.04(0.93,1.16)	1.07(0.95,1.19)	0.0937
Main model	1.00 (reference)	1(0.88,1.13)	1.20(1.04,1.38)^b^	1.35(1.12,1.64)^b^	0.0004^b^
Main model + Nutrients	1.00 (reference)	0.98(0.86,1.12)	1.16(0.99,1.36)	1.27(1.01,1.59)^b^	0.0176^b^
HF incidence					
Minimal model	1.00 (reference)	0.80(0.54,1.18)	0.99(0.68,1.43)	1.15(0.80,1.67)	0.2602
Main model	1.00 (reference)	0.87(0.57,1.33)	1.05(0.65,1.68)	1.16(0.61,2.19)	0.5525
Main model + Nutrients	1.00 (reference)	0.92(0.59,1.43)	1.21(0.72,2.04)	1.48(0.71,3.11)	0.2624
MI incidence					
Minimal model	1.00 (reference)	1.02(0.87,1.21)	0.90(0.76,1.07)	0.92(0.77,1.09)	0.1712
Main model	1.00 (reference)	1.13(0.93,1.37)	1.03(0.82,1.29)	1.07(0.78,1.46)	0.8730
Main model + Nutrients	1.00 (reference)	1.07(0.88,1.30)	0.92(0.72,1.17)	0.86(0.60,1.23)	0.3125
All-cause mortality					
Minimal model	1.14 (1.03,1.27)	1.00 (reference)	1.00(0.89,1.11)	1.01(0.91,1.14)	——^c^
Main model	0.98(0.89,1.09)	1.00 (reference)	1.13(1.02,1.24)^b^	1.22(1.08, 1.39)^b^	——^c^
Main model + Nutrients	1.03(0.91,1.17)	1.00 (reference)	1.04(0.92,1.18)	1.03(0.86,1.25)	——^c^
CVD mortality					
Minimal model	1.34(1.13,1.63)	1.00 (reference)	1.15(0.95,1.39)	1.16(0.96,1.41)	——^c^
Main model	1.16(0.94,1.43)	1.00 (reference)	1.26(1.02,1.54)^b^	1.30(0.99,1.70)	——^c^
Main model + Nutrients	1.15(0.92,1.43)	1.00 (reference)	1.27(1.02,1.57)^b^	1.31(0.96,1.80)	——^c^

^a^Minimal model: Adjusted for age, sex, and study center (as random effect); Main model: Minimal model + demographic data (including location(urban/rural), education, occupation, and marriage), the lifestyle factors (including smoking status, alcohol use, and physical activity), baseline health conditions (including BMI, history of diabetes, hypertension, and CVDs) and total energy intake; Main model + Nutrients: Main model + protein, lipid, carbohydrate, fiber, zinc, iron, sodium, potassium, and magnesium;

^b^*P* < 0.05;

^c^P for trend test was not performed due to the nonlinear trend in the Restricted cubic spline plots;

^d^*CVD* Cardiovascular disease, *HF* Heart failure, *MI* Myocardial infarction, cholesterol

Interaction analysis indicated that location and hypertension play a role in the impact of dietary copper intake on cardiovascular events and mortality risk (P for interaction < 0.05). Stratified analyses were performed to further assess the relations of energy-adjusted dietary copper intake with health in various subgroups. We found stronger association between copper intake and primary outcome among urban residence and individuals with history of hypertension. Similar patterns were observed for secondary outcomes. Besides, dietary copper had a more significant impact on composite outcomes, CVD events, and stroke in the central population (as shown in Supplemental eFigure 2–8). After excluding people with a history of CVD, people with hypertension, diabetes, as well as a history of CVD or individuals who had outcomes of interest in the first 2 years of follow-up, findings remained almost unchanged (as shown in eTable 1 in Additional file 1).

## Discussion

Our prospective cohort study based on a larger population and showed novel evidence that increasing dietary copper intake was associated with a higher risk of all-cause mortality, CVD mortality, CVD incidence, stroke incidence, and the composite outcomes.

Previous epidemiological studies on the association between dietary copper intake and CVD incidence were mostly conducted in the United States [11–13, 15, 16]. A case–control study based on the NHANES showed that the group with the highest dietary copper intake had a significantly lower risk of stroke [11]. The RCS model in this study showed an L-shaped nonlinear relationship between copper intake and stroke, whereas our study showed a higher risk of stroke when dietary copper intake was greater than 2.45mg/d. Such inconsistent results with our study also appear in the relationship between dietary copper intake and MI and total CVD incidence in the NHANES population [12, 13]. The possible explanation for these disparities is the variations in dietary copper intake of the two study populations (2.45 (IQR:1.85–3.19) mg/d in our study vs 1.072 (IQR: 0.799–1.42) mg/d in the NHANES population).

For all-cause and CVD deaths, results from previous studies have been inconsistent. The Japan Collaborative Cohort Study for Evaluation of Cancer Risk (JACC) found that compared with the lowest quintiles of copper intake, the multivariable HRs for CVD mortality in the highest quintiles were 1.63 (95%CI: 1.21–2.33) among men and 1.36 (95%CI: 1.06–1.69) among women [15]. Positive associations between dietary copper intake and death from stroke and other CVDs have also been found in this study. LASSO penalty regression analysis and multi-metal model were used to analyze the association between basic metal levels and all-cause mortality and CVD mortality in patients with diabetes in Dongfeng-Tongji cohort. They reported a J-shape relationship between copper intake and all-cause mortality [28]. The CHNS study, a Chinese prospective cohort with a median dietary copper intake of 1.83 (IQR:1.60–2.09)mg/d, reported that when dietary copper intake was lower than 2.09 mg/d, the risk of all-cause mortality tended to be stable, and once dietary copper intake exceeded this value, the risk of death would increase significantly [16]. British National Diet and Nutrition Survey in which the dietary copper intake is reported to be 1.10 ± 0.68 mg/d for males and 0.88 ± 0.55 mg/d in females showed that higher dietary intakes of copper were associated with lower total mortality (HR:0.91; 95% CI:0.84, 1.00) [17]. Significant regional differences in dietary copper intake were observed in these cohorts: Asian countries such as China and Japan have much higher dietary copper intake than European and American regions such as the United States and the United Kingdom. This may be caused by different dietary habits between Eastern and Western countries, with the Chinese diet tending to contain more cop-rich grains and legumes, while the Western diet is dominated by meat with relatively low copper content [29, 30]. This phenomenon provides evidence for the different findings in different countries and suggest that moderate copper intake may reduce cardiovascular health risks and excessive copper intake may be harmful to our health.

The cardiovascular health effects of dietary copper intake are related to its role in the human body. It has been proven that copper in the human body plays a central role in the strength and integrity of the heart and blood vessels [31, 32]. Cuproptosis can lead to the development of CVDs such as atherosclerosis, stroke, ischemic heart disease and HF [31, 33–35]. In addition, copper deficiency has been suggested to be associated with a higher risk of acute MI [36]. Therefore, the risk of CVD can only be effectively reduced if copper levels are controlled below toxic doses and above deficient doses. However, our study did not observe a statistically significant association among those with lower dietary copper intake. Based on previous results obtained in populations with low dietary copper intake [11–13, 17], this may be due to the generally high dietary copper intake in the PURE China cohort, which did not reach the range of copper deficiency.

Disparities exist in the recommended dietary copper intake value across the world. WHO/FAO estimates that the daily dietary requirement for copper is 0.7 mg/d for women and 0.8 mg/d for men. Most developed countries set the recommended dietary copper intake for adults as 0.900–1.7 mg/d for men and 0.9–1.5 mg/d for women [37]. According to the Chinese Nutrient Dietary Reference Intake (DRIs), the dietary reference intake (RNI) of copper for adult aged 18–64 years should be 0.8 mg/d, and the tolerable upper intake level is 8 mg/d. Our study indicated that the current dietary copper intake of the Chinese population is about 2.6 ± 1.1 mg/d, and 75% of the population intake is 1.85–3.19 mg/d, which is much higher than the recommended intake. Within this range, increased copper intake may increase the risk of CVDs. Therefore, recommendations for the tolerable upper intake level of dietary copper may need to be stricter.

Our research still has some limitations. First, the diet questionnaire used to calculate dietary copper intake was collected through self-report, which could have resulted in some recall bias. However, previous studies have shown similar results with our study [11, 13, 16, 28]. And FFQ is still one of the most commonly used methods in current dietary nutrition surveys [38–40]. Moreover, the FFQ we use has been proven to have sufficient reproducibility and validity [25]. Besides, even for the same food, copper content may be different due to different species and regions and cannot accurately reflect the actual copper intake. The current study calculated dietary copper intake based on the China Food Composition Table (2002), which represented the national average level. Moreover, due to the limitations of current data, our study used only baseline copper intake as an estimate of individual dietary copper intake, leading to a failure in capturing dietary changes over time. Finally, due to the high dietary copper intake in our study population, the results of this study reflect the cardiovascular health effects of copper excess. Previous epidemiological studies also have such lopsided results due to the overall high or low dietary intake of the study population [11, 13, 15, 16]. Therefore, more extensive epidemiological studies are still needed to determine the optimal dietary copper intake.

## Conclusions

Our study based on a large population-based prospective cohort study demonstrated that higher dietary copper intake was associated with a higher risk of composite outcomes, CVDs, and stroke, all-cause death and CVD death. The results of this study revealed that the current dietary copper intake of the Chinese population was higher than the dietary reference intake, and the population intake level of copper from diet is suggested lowering down to a relatively safety threshold for mitigating the increasing burden of CVD among Chinese population. However, more studies are needed to verify the ideal dietary copper intake.

### Supplementary Information

**Additional file 1. **

**Additional file 2. **

## Data Availability

The datasets analyzed during the current study are not publicly available due to an ongoing project, but are available from the corresponding author on reasonable request.
