# Supplementary material for: Associations of dietary copper intake with cardiovascular disease and mortality: findings from the Chinese Perspective Urban and Rural Epidemiology (PURE-China) Study
Source: BMC Public Health. 2023 Dec 18;23:2525. doi: 10.1186/s12889-023-17441-6 (PMC10726617; doi:10.1186/s12889-023-17441-6)
Supplement: Supplementary file 1 — Additional file 1. [file 12889_2023_17441_MOESM1_ESM.docx]

**ELEMENT LIST**

**eTable 1** Sensitivities analyses for the associations between dietary Cu intake and health outcomes.

**eFigure 1** Restricted cubic spline plots between dietary copper intake with death and CVDs.

**eFigure 2** Stratified analyses by potential effect modifiers for the associations between dietary Cu intake and the risk of composite outcomes in various subgroups.

**eFigure 3** Stratified analyses by potential effect modifiers for the associations between dietary Cu intake and the risk of CVD incidence in various subgroups.

**eFigure 4** Stratified analyses by potential effect modifiers for the associations between dietary Cu intake and the risk of Stroke incidence in various subgroups.

**eFigure 5** Stratified analyses by potential effect modifiers for the associations between dietary Cu intake and the risk of MI incidence in various subgroups.

**eFigure 6** Stratified analyses by potential effect modifiers for the associations between dietary Cu intake and the risk of HF incidence in various subgroups.

**eFigure 7** Stratified analyses by potential effect modifiers for the associations between dietary Cu intake and the risk of all-cause mortality in various subgroups.

**eFigure 8** Stratified analyses by potential effect modifiers for the associations between dietary Cu intake and the risk of CVD mortality in various subgroups

eTable 1 Sensitivities analyses for the associations between dietary Cu intake and health outcomes.

|  | Dietary copper intake | | | |
| --- | --- | --- | --- | --- |
|  | <1.85 mg/d | 1.85-2.45 mg/d | 2.45-3.19 mg/d | >3.19 mg/d |
| Excluding people with a history of CVD | | | | |
| Composite outcome | 1.00 (reference) | 1.00(0.91,1.11) | 1.13(1.01,1.27) | 1.22(1.04,1.41) |
| CVD incidence | 1.00 (reference) | 1.01(0.89,1.13) | 1.17(1.02,1.34) | 1.27(1.06,1.52) |
| Stroke incidence | 1.00 (reference) | 0.99(0.86,1.14) | 1.21(1.03,1.41) | 1.36(1.1,1.68) |
| HF incidence | 1.00 (reference) | 0.65(0.38,1.11) | 0.99(0.56,1.72) | 1.07(0.51,2.27) |
| MI incidence | 1.00 (reference) | 1.10(0.88,1.37) | 1.08(0.83,1.40) | 1.12(0.78,1.60) |
| All-cause mortality | 1.04(0.90,1.19) | 1.00 (reference) | 1.12(0.98,1.28) | 1.14(0.96,1.36) |
| CVD mortality | 1.22(0.95,1.56) | 1.00 (reference) | 1.38(1.09,1.75) | 1.35(0.99,1.85) |
| Excluding people with hypertension, diabetes and a history of CVD | | | | |
| Composite outcome | 1.00 (reference) | 1.01(0.93,1.11) | 1.1(0.99,1.22) | 1.17(1.01,1.35) |
| CVD incidence | 1.00 (reference) | 1.00(0.90,1.12) | 1.14(1.01,1.29) | 1.25(1.05,1.48) |
| Stroke incidence | 1.00 (reference) | 0.98(0.86,1.11) | 1.20(1.04,1.39) | 1.34(1.10,1.63) |
| HF incidence | 1.00 (reference) | 0.73(0.47,1.15) | 0.91(0.56,1.48) | 1.01(0.52,1.95) |
| MI incidence | 1.00 (reference) | 1.15(0.94,1.40) | 1.05(0.83,1.33) | 1.15(0.83,1.59) |
| All-cause mortality | 1.01(0.89,1.15) | 1.00 (reference) | 1.06(0.93,1.2) | 1.09(0.92,1.29) |
| CVD mortality | 1.16(0.93,1.45) | 1.00 (reference) | 1.27(1.02,1.57) | 1.36(1.03,1.81) |
| Excluding patients with an outcome within 2 years | | | | |
| Composite outcome | 1.00 (reference) | 1.01(0.93,1.11) | 1.10(0.99,1.22) | 1.17(1.01,1.35) |
| CVD incidence | 1.00 (reference) | 1.00(0.90,1.12) | 1.14(1.01,1.29) | 1.25(1.05,1.48) |
| Stroke incidence | 1.00 (reference) | 0.98(0.86,1.11) | 1.20(1.04,1.39) | 1.34(1.10,1.63) |
| HF incidence | 1.00 (reference) | 0.73(0.47,1.15) | 0.91(0.56,1.48) | 1.01(0.52,1.95) |
| MI incidence | 1.00 (reference) | 1.15(0.94,1.40) | 1.05(0.83,1.33) | 1.15(0.83,1.59) |
| All-cause mortality | 1.01(0.89,1.15) | 1.00 (reference) | 1.06(0.93,1.2) | 1.09(0.92,1.29) |
| CVD mortality | 1.16(0.93,1.45) | 1.00 (reference) | 1.27(1.02,1.57) | 1.36(1.03,1.81) |

eFigure 1 Restricted cubic spline plots between dietary copper intake with death and CVDs.


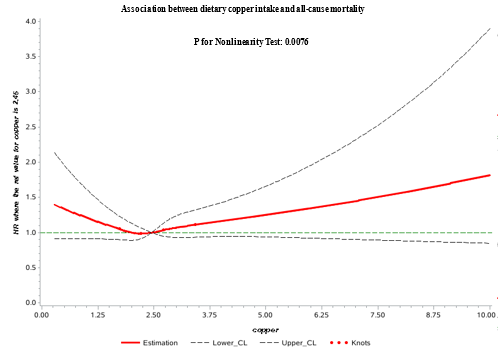

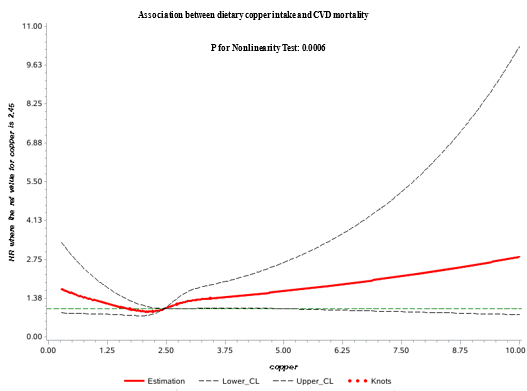

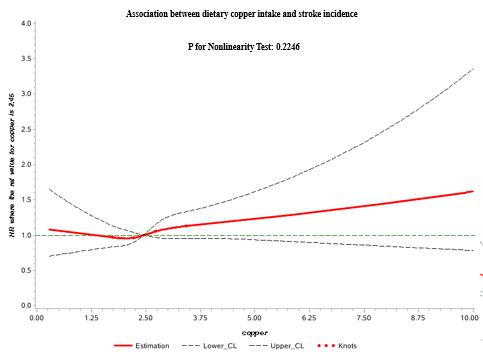

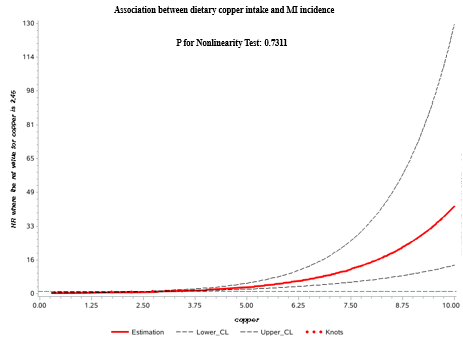

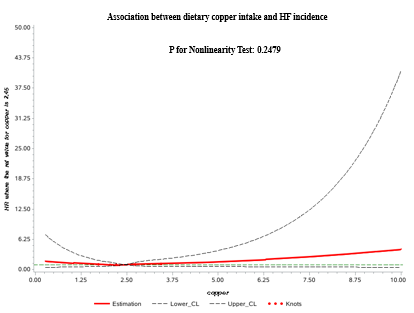

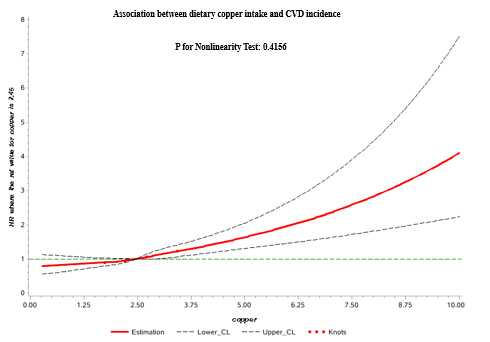


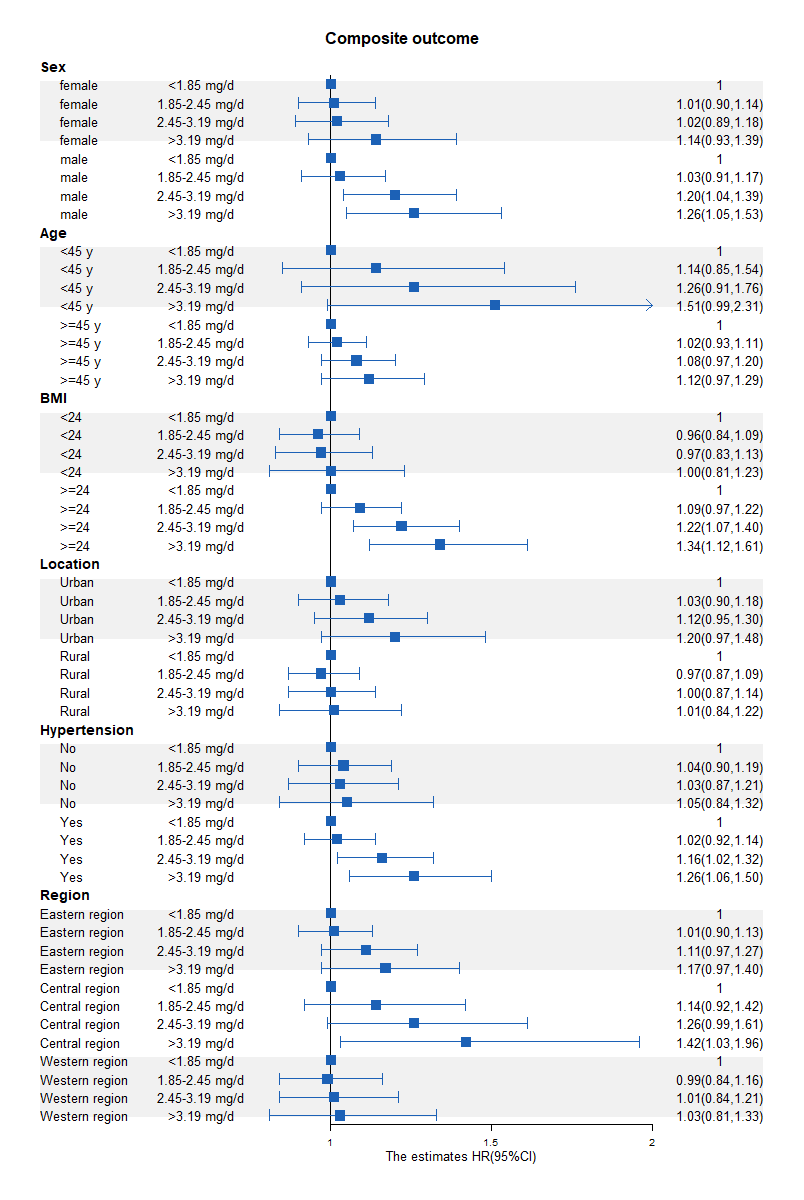


eFigure 2 Stratified analyses by potential effect modifiers for the associations between dietary Cu intake and the risk of composite outcomes in various subgroups.


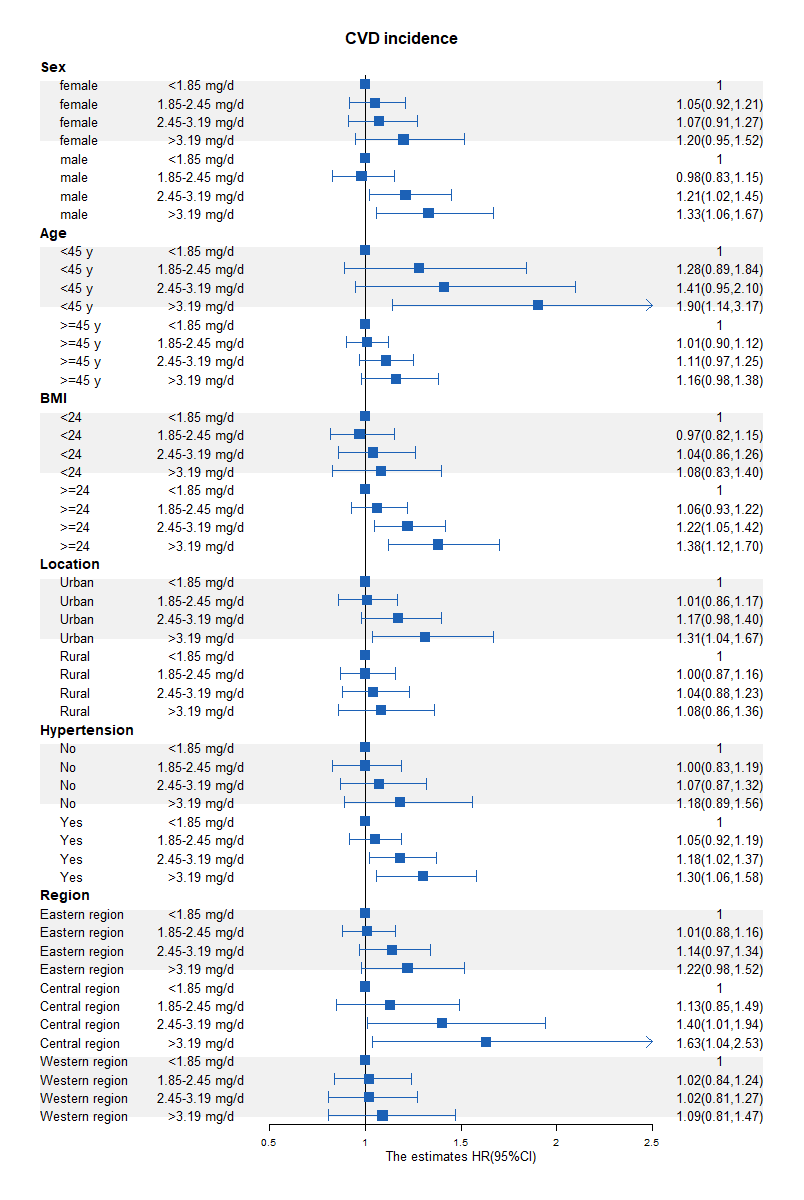


eFigure 3 Stratified analyses by potential effect modifiers for the associations between dietary Cu intake and the risk of CVD incidence in various subgroups.


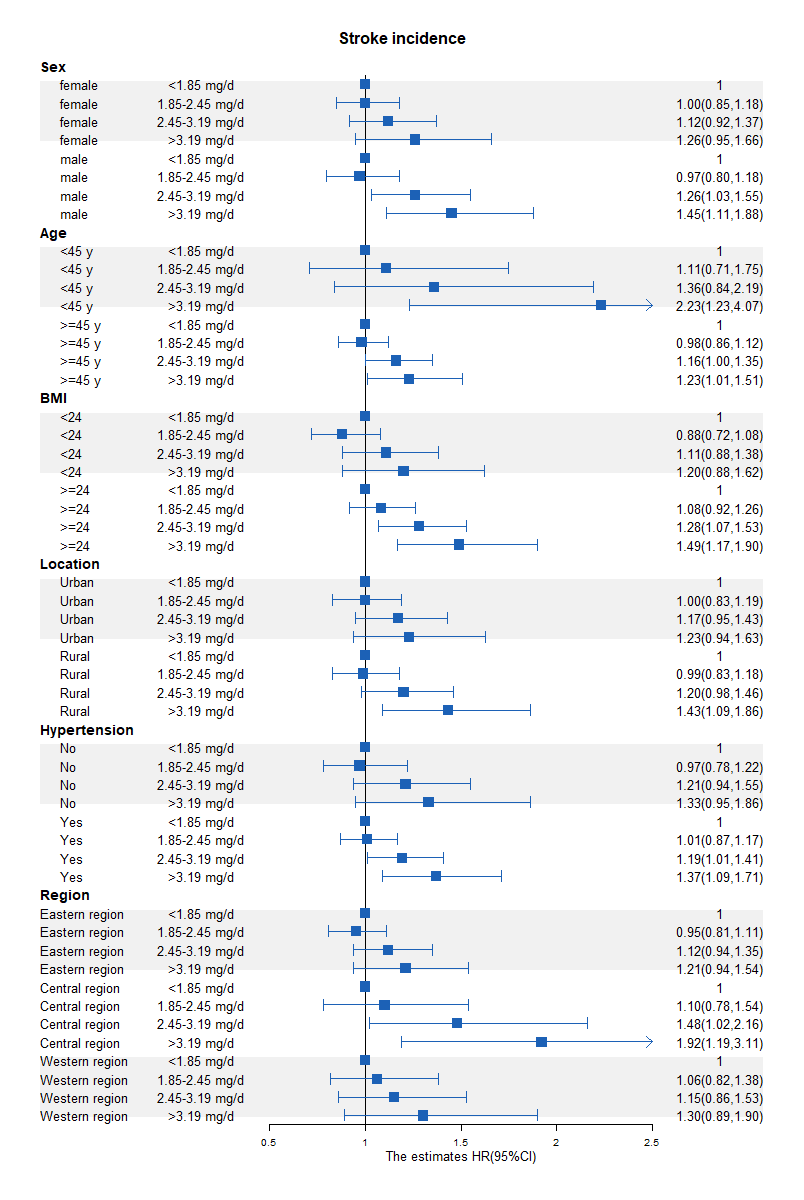
 eFigure 4 Stratified analyses by potential effect modifiers for the associations between dietary Cu intake and the risk of Stroke incidence in various subgroups.


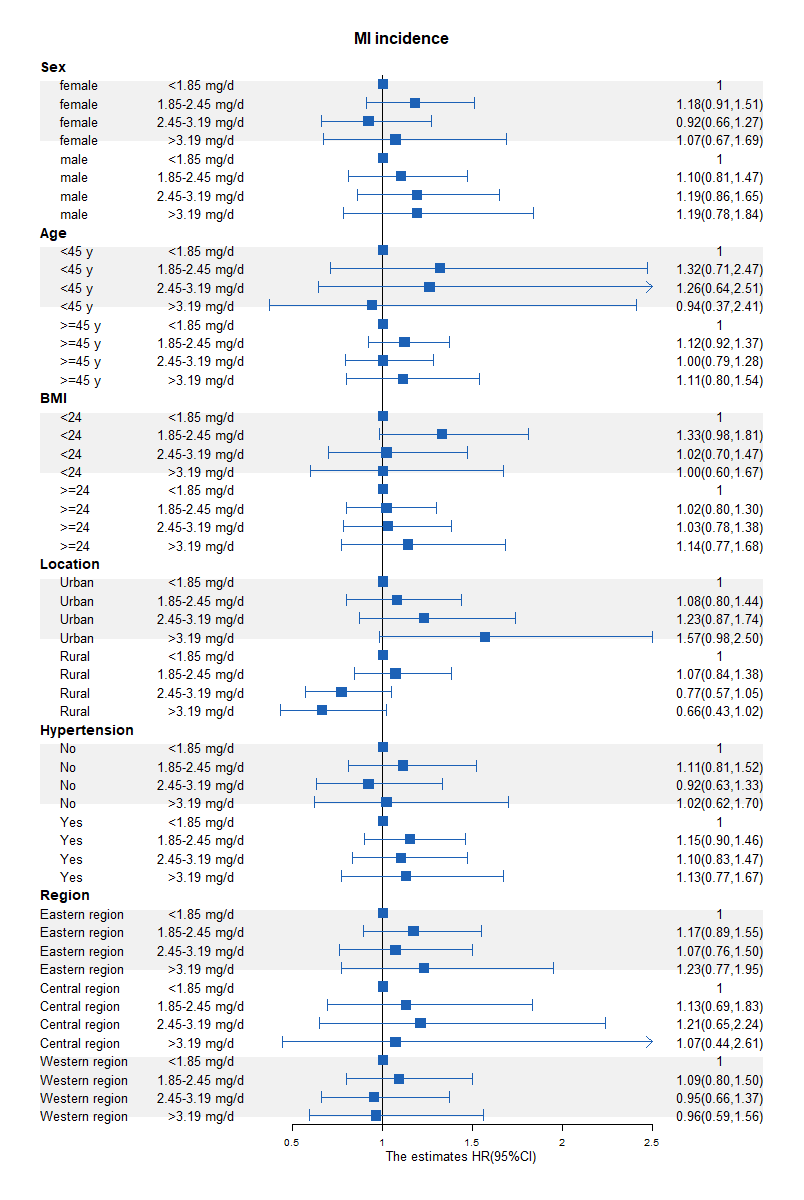


eFigure 5 Stratified analyses by potential effect modifiers for the associations between dietary Cu intake and the risk of MI incidence in various subgroups.


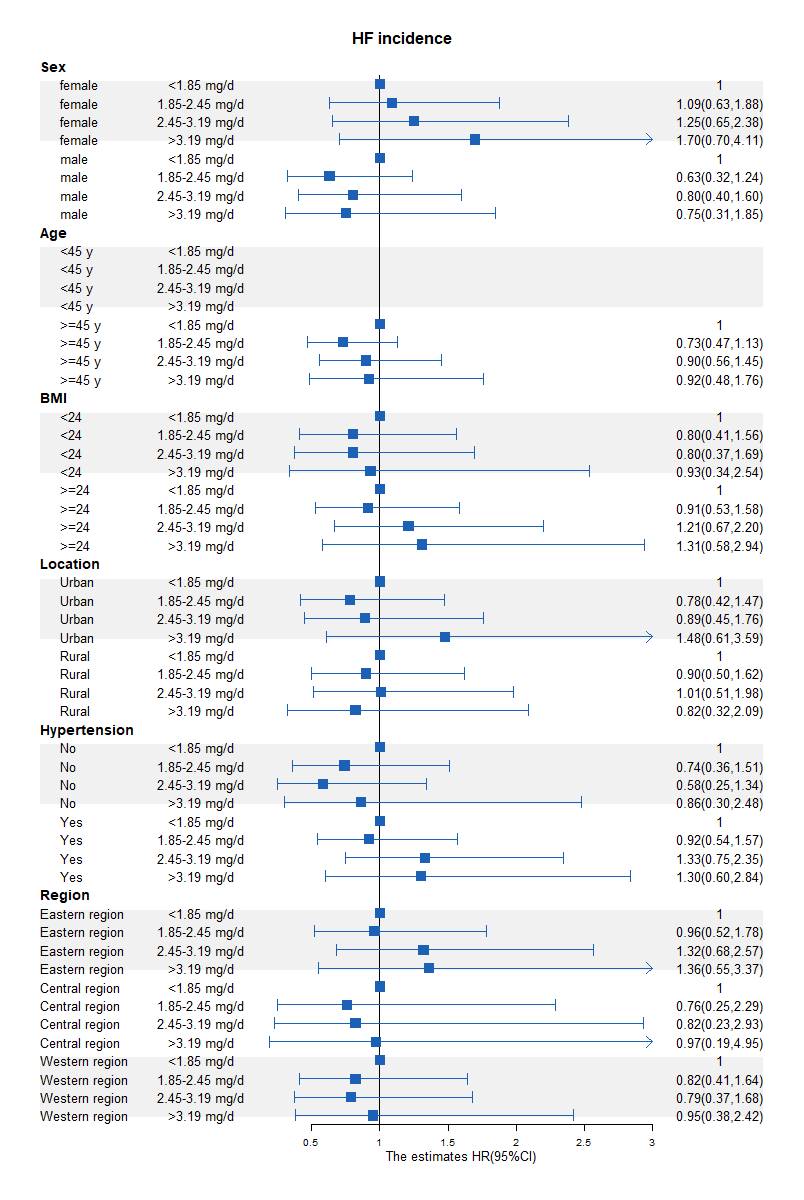


eFigure 6 Stratified analyses by potential effect modifiers for the associations between dietary Cu intake and the risk of HF incidence in various subgroups.
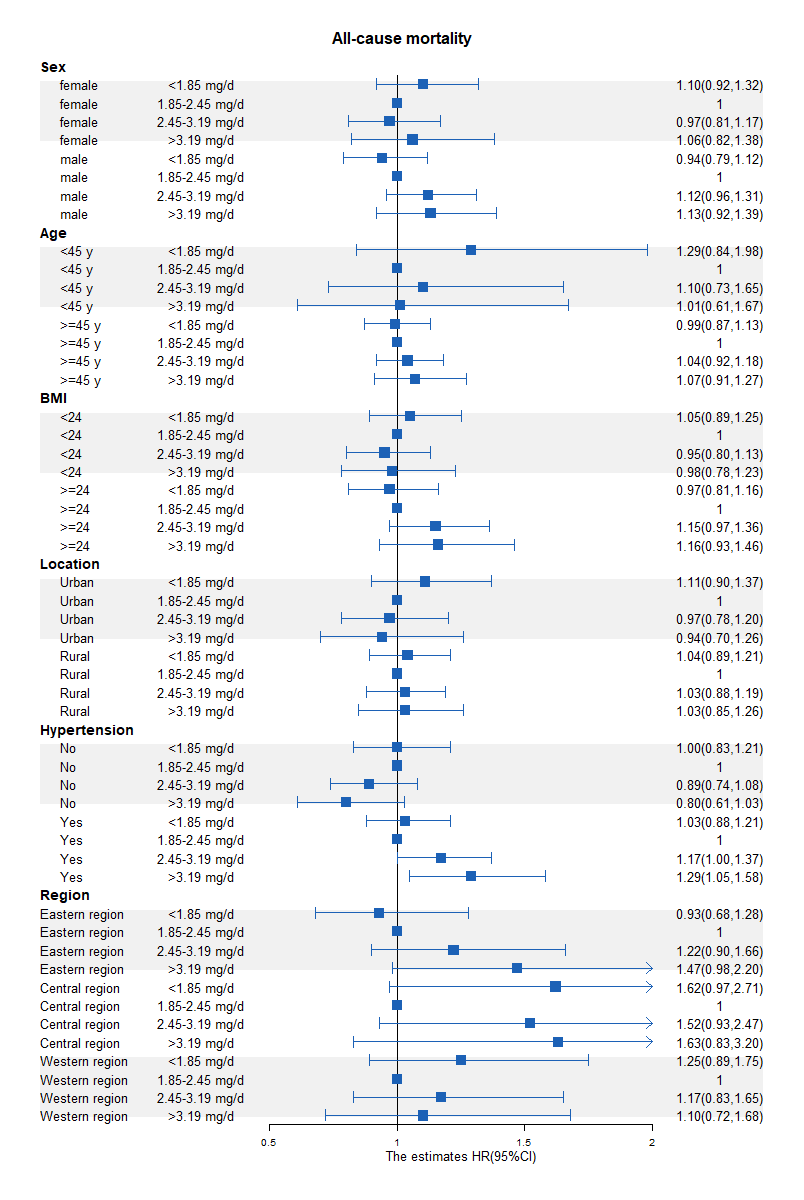


eFigure 7 Stratified analyses by potential effect modifiers for the associations between dietary Cu intake and the risk of all-cause mortality in various subgroups.


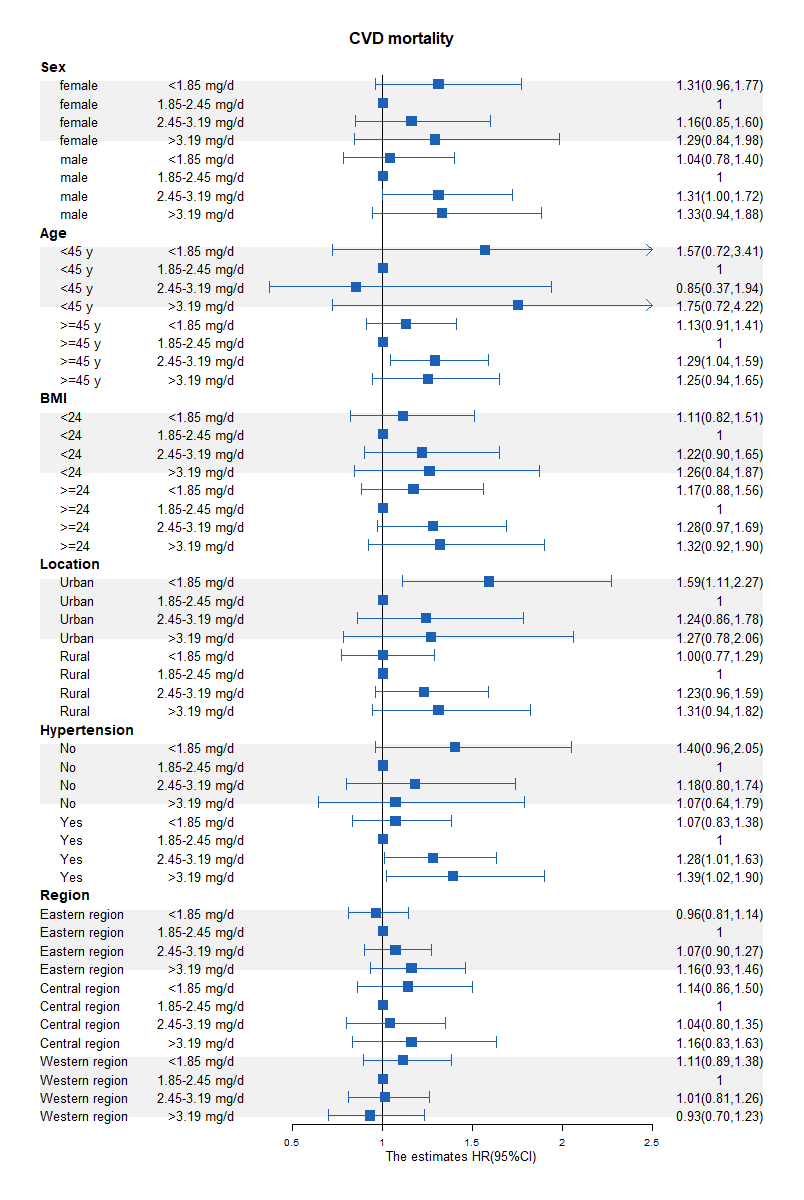
 eFigure 8 Stratified analyses by potential effect modifiers for the associations between dietary Cu intake and the risk of CVD mortality in various subgroups
