# Supplementary material for: Associations of dietary copper intake with cardiovascular disease and mortality: findings from the Chinese Perspective Urban and Rural Epidemiology (PURE-China) Study
Source: BMC Public Health. 2023 Dec 18;23:2525. doi: 10.1186/s12889-023-17441-6 (PMC10726617; doi:10.1186/s12889-023-17441-6)
Supplement: Supplementary file 2 — Additional file 2. [file 12889_2023_17441_MOESM2_ESM.docx]

**Event Definitions**

**FATAL EVENTS**

**Cardiovascular Death – Definitions**

**01.00 DEATH DUE TO CARDIOVASCULAR EVENTS**

**01.10 Sudden unexpected Cardiovascular Death (SCVD)**

Without evidence of other cause of death, death that occurred suddenly and unexpectedly (examples: witnessed collapse, persons resuscitated from cardiac arrest who later died) or persons seen alive less than 12 hours prior to discovery of death (example persons found dead in his/her bed).

SCVD is either definite, probable or possible according to the following characteristics:

| **PURE Adjudication Code** | **Event Type** | **Event Type** |
| --- | --- | --- |
| **01.11: Definite** | One of the following in persons with:  • known cardiovascular disease, or  • diabetes with an additional risk factor such as hypertension, smoking, dyslipidemia, micro albuminuria, serum creatinine 50% above upper limit of normal, or  • 3 of the above risk factors, or  • 2 of the above risk factors in men aged 60 and more and women aged 65 and more | No ICD-10 Code |
| **01.12: Probable** | One of the following in persons with:  • diabetes, or  • 2 of the above risk factors in men aged less than 60 and in women less than 65, or  • one of the above risk factor in men aged 60 and more and in women aged 65 and more, or  • typical of chest pain or sudden severe dyspnea of less than 20-minute duration preceding the event |  |
| **01.13: Possible** | In persons without risk factor |  |
| For **SCVD**, the patient was well or had a stable CVD (example stable angina) when last seen alive. The event of a sudden death occurring during the hospitalization of MI is considered a fatal MI and not sudden death. | | |

**01.30 Fatal Myocardial Infarction**

**Symptoms of Myocardial Infarction:**

Typical symptoms or suggestive symptoms of MI according to physician are characterized by severe anterior chest pain as tightness, crushing, burning, lasting at least 20 minutes, occurring at rest, or on exertion, that may radiate to the arms or neck or jaw and may be associated with dyspnea, diaphoresis and nausea. However, death associated with nausea and vomiting with or without chest pain not due to another cause may be considered as possible MI if ECG and cardiac markers are not done. These symptoms may have occurred the last month before death.

Fatal myocardial infarction is either definite, probable or possible according to the following characteristics:

| **PURE Adjudication Code** | **Event Type** | **Event Type** |
| --- | --- | --- |
| **01.31: Definite** | 1. Autopsy demonstrating fresh myocardial infarction and/or recent coronary occlusion, or  2. ECG showing new and definite sign of MI (Minnesota code 1-1-1) or  3. Symptoms typical or atypical or inadequately described but attributed to cardiac origin lasting at least 20 minutes and by troponin or cardiac enzymes (CKMB, CK, SGOT, SLDH) above center laboratory ULN  4. ECG with new ischemic changes (new ST elevation/depression or T wave inversion ≥ 2 mm) and by troponin or cardiac enzymes (CKMB, CK, SGOT, SLDH) above center laboratory ULN | I21-I22 |
| **01.32: Probable** | 1. ECG with sign of probable MI (Minnesota code 1-2-1), or  2. Typical symptoms lasting at least 20 minutes considered of cardiac origin, with only new ST-T changes (new ST elevation/depression or T wave inversion ≥ 1 but < 2mm) without documented increased cardiac markers or enzyme as in PURE definition 1.31 (above), or  3. Increased cardiac enzymes as in PURE definition 1.31 (above) showing a typical pattern of MI as above without symptoms or significant ECG changes |  |
| **01.33: Possible** | 1. ECG with sign of possible MI (Minnesota code 1-3-1) or  2. Typical symptoms or symptoms suggestive of MI according to the physician lasting at least 20 minutes without documented ECG or cardiac marker. |  |

The Minnesota codes for MI is taken from Rose and Blackburn and published in their book “Evaluation Methods of Cardiovascular Disease WHO 1969”.

- Definite MI is Q/R ratio ≥1/3 and Q duration ≥ 0.03 second in one of the following leads: I, II, V2, 3, 4, 5, 6. (code 1-1-1)
- Probable MI is Q/R ratio ≥1/3 and Q duration between 0.02 and 0.03 second in one of the following leads: I, II, V2, 3, 4, 5, 6. (code 1-2-1)
- Possible MI is Q/R ratio between 1/5 and 1/3 and Q duration between 0.02 and 0.03 second in one of the following leads: I, II, V2, 3, 4, 5, 6. (code 1-3-1)

**01.40 Fatal Stroke**

Fatal stroke is either definite or possible according to the following characteristics:

| **PURE Adjudication Code** | **Event Type** | **Event Type** |
| --- | --- | --- |
| **01.41: Definite** | Stroke death is defined as death within 30 days from an acute focal neurological deficit diagnosed by a physician and thought to be of vascular origin (without other cause such as brain tumor) with signs and symptoms lasting >= 24 hrs.  Stroke death is also considered if death occurred within 24 hrs. of onset of persisting signs and symptoms, or if there is evidence of a recent stroke on autopsy.  N.B.   - **In a subject with a stroke <= 30 days:** If death occurred with a pneumonia due to possible aspiration, death will be considered to be due to stroke. - **In a subject with a stroke > 30 days:** If death occurred with a pneumonia due to possible aspiration, the adjudicator will make a decision according to his/her clinical judgment if death is related to stroke or not. - Subarachnoid hemorrhage death manifested by sudden onset headache with/without focal signs and imaging (CT or MRI) evidence of bleeding primarily in the subarachnoid space is considered a fatal stroke in absence of trauma or brain tumor or malformation - Subdural hematoma death is not considered as a stroke death and may be related to previous trauma or other cause | I60-I64, I69 |
| **01.43: Possible** | Death in a participant with a history of sudden onset of  focal neurological deficit of one or more limbs, loss of  vision or slurred speech lasting about 24 hours. |  |

**01.50 Fatal Congestive Heart Failure**

Fatal congestive heart failure is either definite or possible according to the following characteristics:

| **PURE Adjudication Code** | **Event Type** | **Event Type** |
| --- | --- | --- |
| **01.51: Definite** | The diagnosis of congestive heart failure may be an autopsy finding in absence of other cause or requires signs (rales, increased jugular venous pressure or ankle edema) or symptoms (nocturnal paroxysmal dyspnea, dyspnea at rest or ankle edema) of congestive heart failure and one or both of the following:   - radiological signs of pulmonary congestion, - treatment of heart failure with diuretics   *If sudden death occurred in a patient with chronic severe heart failure, it should be adjudicated as fatal congestive heart failure.* | I50 |
| **01.52: Probable** | Progressive shortness of breath on lying down or at night, improving on sitting up AND any of the following signs or symptoms: swelling of feet, distension of abdomen, progressive cough in a person with known hypertension or a history of previous MI/angina or other heart disease |  |
| **01.53: Possible** | Progressive shortness of breath on lying down or at night, improving on sitting up AND any of the following signs or symptoms: swelling of feet, distension of abdomen,  progressive cough |  |

**01.60 Death Due to Other Cardiovascular Deaths** (other causes [1.10 to 1.50 above] having been excluded)

| **PURE Adjudication Code** | **Event Type** | **Event Type** |
| --- | --- | --- |
| **01.61** | Arterial rupture of aneurysm | I71- I72 |
| **01.62** | Pulmonary embolism  NOTE: Death associated with pulmonary embolism occurring **within 2 weeks** after a fracture such as hip, femur should attributed to death due to injury. Refer to Injury, Section 6.0 | I26 |
| **01.63** | Arrhythmic death (A-V block, sustained ventricular tachycardia in absence of other causes) | I44-I45, I47-I49 |
| **01.64** | Death after invasive cardiovascular intervention: a perioperative death extending to 30 days after coronary or arterial surgical revascularization and to 7 days after a coronary or arterial percutaneous dilatation (angioplasty) with or without a stent or an invasive diagnostic procedure. | I97 |
| **01.65** | Congenital heart disease | Q20-Q28 |
| **01.66** | Heart valve disease (including rheumatic heart disease) | I01, I05-I09, I34-I37 |
| **01.67** | Endocarditis | I33, I38 |
| **01.68** | Myocarditis | I40 |
| **01.69** | Tamponade (pericarditis) | I30, I31, I32 |
| **01.70** | Other cardiovascular events *(Excluding* ***1.61 to 1.69*** *above)*  Valid ICD-10 codes would include the following:  I11, I12, I13, I23, I24, I25, I27, I28, I42, I51, I52, I65-I68, I73, I74, I96, I98, I99 (Refer to ICD-10 Listing for associated definitions for each code) | Any valid ‘I’ (Cardiovascular)  ICD-10 code that can be classified as underlying Code, not specified above |

**NON-FATAL EVENTS**

**Cardiovascular Events – Definitions**

**10.00 NON-FATAL CARDIOVASCULAR EVENTS**

**10.10 Non-Periprocedural Myocardial Infarction (MI)**

MI is considered either definite, probable or possible according to the following characteristics:

| **PURE Adjudication Code** | **Event Type** | **Event Type** |
| --- | --- | --- |
| **10.11: Definite** | 5. ECG showing new and definite sign of MI (Minnesota code 1-1-1) or  6. Symptoms typical or atypical or inadequately described but attributed to cardiac origin lasting at least 20 minutes and by troponin or cardiac enzymes (CKMB, CK, SGOT, SLDH) above center laboratory ULN  7. ECG with new ischemic changes (new ST elevation/depression or T wave inversion ≥ 2 mm) and by troponin or cardiac enzymes (CKMB, CK, SGOT, SLDH) above center laboratory ULN  Please note that increased markers may occur in trauma (CK, AST, myoglobin and CK MB to a lesser degree); renal insufficiency, heart failure, pulmonary embolism…. (troponin), cardioversion (all) | I21-I22 |
| **10.12: Probable** | 4. ECG with new and probable sign of MI (Minnesota code 1-2-1), or  5. Typical symptoms lasting at least 20 minutes considered of cardiac origin, with only new ST-T changes (new ST elevation/depression or T wave inversion ≥ 1 but < 2mm) without documented increased cardiac markers as in PURE definition 10.11 (above), or  6. Increased cardiac enzymes showing a typical pattern of MI as above without symptoms or significant ECG changes |  |
| **10.13: Possible** | 1. ECG with new and possible sign of MI (Minnesota code 1-3-1), or  2. Typical symptoms lasting 20 minutes and more considered to be of cardiac origin without documented ECG or cardiac marker. |  |

**10.20 Periprocedural Myocardial Infarction**

| **PURE Adjudication Code** | **Event Type** | **Event Type** |
| --- | --- | --- |
| **10.21: Definite** | 1. ECG showing new and definite sign of MI (Minnesota code 1-1-1), or  2. Increased cardiac markers within 48 hours of procedure:   - percutaneous coronary intervention: CKMB should be ≥ 5 X ULN or troponin ≥ 5 X above lower level of necrosis OR > 20% increase in cardiac markers if elevated at the beginning of the procedure in a patient with symptoms suggestive of myocardial ischemia - Coronary surgery: Increased cardiac markers CKMB should be ≥ 10X ULN or troponin ≥ 10X above lower limit of necrosis. | I21-I22 |

The **Minnesota codes** for MI is taken from Rose and Blackburn and published in their book “Evaluation Methods of Cardiovascular Disease WHO 1969”.

- **Definite MI** is Q/R ratio ≥1/3 and Q duration ≥ 0.03 second in one of the following leads: I, II, V2, 3, 4, 5, 6. (code 1-1-1)
- **Probable MI** is Q/R ratio ≥1/3 and Q duration between 0.02 and 0.03 second in one of the following leads: I, II, V2, 3, 4, 5, 6. (code 1-2-1)

**Possible MI** is Q/R ratio between 1/5 and 1/3 and Q duration between 0.02 and 0.03 second in one of the following leads: I, II, V2, 3, 4, 5, 6. (code 1-3-1)

**10.30 Stroke/Transient Ischemic Attack (TIA)**

| **PURE Adjudication Code** | **Event Type** | **Event Type** |
| --- | --- | --- |
| **10.31: Definite** | Stroke is defined as an acute focal neurological deficit **diagnosed by a physician** and thought to be of vascular origin (without other case such as brain tumor) with signs and symptoms lasting ≥ 24 hrs.  N.B.   - Subarachnoid hemorrhage manifested by sudden onset headache with/without focal signs and imaging (CT or MRI or lumbar puncture) showing evidence of bleeding primarily in the subarachnoid space is considered a stroke in absence of trauma or brain tumor or malformation - Subdural hematoma is not considered as a stroke and may be related to previous trauma or other cause. | I60-I64, I69 |
| **10.33: Possible** | Stroke is possible if there is a history of sudden onset of focal neurological deficit of one or more limbs, loss of vision or slurred speech lasting about 24 hours or more |  |
| **10.34: TIA** | The diagnosis of TIA requires the presence of acute focal neurological deficit thought to be of vascular origin with signs and symptoms lasting less than 24 hours | G45 |

**10.40 Congestive Heart Failure**

| **PURE Adjudication Code** | **Event Type** | **Event Type** |
| --- | --- | --- |
| **10.41: Definite** | The diagnosis of congestive heart failure requires signs (rales, increased jugular venous pressure or ankle edema) or symptoms (nocturnal paroxysmal dyspnea, dyspnea at rest or ankle edema) of congestive heart failure and one or both of the following:   - radiological signs of pulmonary congestion, - Treatment of heart failure with diuretics. | I50 |
| **10.42: Probable** | Progressive shortness of breath on lying down or at night, improving on sitting up AND any of the following signs or symptoms: swelling of feet, distension of abdomen, progressive cough in a person with known hypertension or a history of previous MI/angina or other heart disease |  |
| **10.43: Possible** | Congestive heart failure is considered possible when there is progressive shortness of breath on lying down or at night, improving on sitting up AND any of the following signs or symptoms: swelling of feet, distension of abdomen, progressive cough |  |
